# FGF23 Actions on Target Tissues—With and Without Klotho

**DOI:** 10.3389/fendo.2018.00189

**Published:** 2018-05-02

**Authors:** Beatrice Richter, Christian Faul

**Affiliations:** Division of Nephrology, Department of Medicine, The University of Alabama at Birmingham, Birmingham, AL, United States

**Keywords:** FGF23, klotho, fibroblast growth factor receptor 4, chronic kidney disease, cardiac hypertrophy, inflammation

## Abstract

Fibroblast growth factor (FGF) 23 is a phosphaturic hormone whose physiologic actions on target tissues are mediated by FGF receptors (FGFR) and klotho, which functions as a co-receptor that increases the binding affinity of FGF23 for FGFRs. By stimulating FGFR/klotho complexes in the kidney and parathyroid gland, FGF23 reduces renal phosphate uptake and secretion of parathyroid hormone, respectively, thereby acting as a key regulator of phosphate metabolism. Recently, it has been shown that FGF23 can also target cell types that lack klotho. This unconventional signaling event occurs in an FGFR-dependent manner, but involves other downstream signaling pathways than in “classic” klotho-expressing target organs. It appears that klotho-independent signaling mechanisms are only activated in the presence of high FGF23 concentrations and result in pathologic cellular changes. Therefore, it has been postulated that massive elevations in circulating levels of FGF23, as found in patients with chronic kidney disease, contribute to associated pathologies by targeting cells and tissues that lack klotho. This includes the induction of cardiac hypertrophy and fibrosis, the elevation of inflammatory cytokine expression in the liver, and the inhibition of neutrophil recruitment. Here, we describe the signaling and cellular events that are caused by FGF23 in tissues lacking klotho, and we discuss FGF23’s potential role as a hormone with widespread pathologic actions. Since the soluble form of klotho can function as a circulating co-receptor for FGF23, we also discuss the potential inhibitory effects of soluble klotho on FGF23-mediated signaling which might—at least partially—underlie the pleiotropic tissue-protective functions of klotho.

## FGF23—A Brief Introduction

The family of fibroblast growth factors (FGF) consists of 22 members in humans, with a broad range of biological functions, including the regulation of embryonic development, organogenesis, and metabolism (1). FGFs are divided into seven subfamilies based on phylogenic analyses and overlapping structures (2). Members of the FGF19 subfamily, consisting of FGF19, FGF21, and FGF23, function as circulating hormones and are, therefore, termed endocrine FGFs (3, 4). Unlike paracrine FGFs, such as FGF1 or FGF2, endocrine FGFs share a characteristic structure and lack the heparin-binding domain in their C-terminus which enables their secretion, circulation, and action on distant target organs (5, 6).

**Figure 1:**
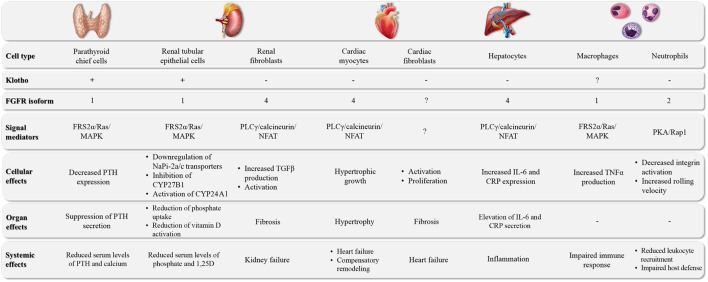
The major target organs of FGF23. FGF23 can directly target different cell types in a variety of organs. The underlying molecular pathways can differ in their requirement for klotho as well as the involvement of specific FGFR isoforms and downstream signal mediators, leading to cell type-specific events and tissue-specific effects.

FGF23 is a bone-derived hormone that lowers serum phosphate levels (7–9). Dietary phosphate intake stimulates the production and secretion of FGF23 from osteocytes, and FGF23 directly targets the kidney to increase phosphate excretion by downregulating the cell surface expression of the sodium-dependent phosphate transporters, NaPi-2a and NaPi-2c, in the proximal tubule (10–14) (Figure 1). In addition, FGF23 reduces circulating levels of active vitamin D by inhibiting renal 1-α-hydroxylase (also called CYP27B1), the enzyme that converts the prehormone 25-hydroxyvitamin D into its active form, 1,25-dihydroxyvitamin D (1,25D), and by increasing the expression of 24-hydroxylase (also called CYP24A1), the enzyme that degrades 1,25D into inactive metabolites (10–14). In the parathyroid gland, FGF23 inhibits the secretion of parathyroid hormone (PTH) (15, 16) (Figure 1). FGF23’s effects on reducing circulating levels of 1,25D and PTH further contribute to its phosphaturic actions (3, 17).

**Figure 2:**
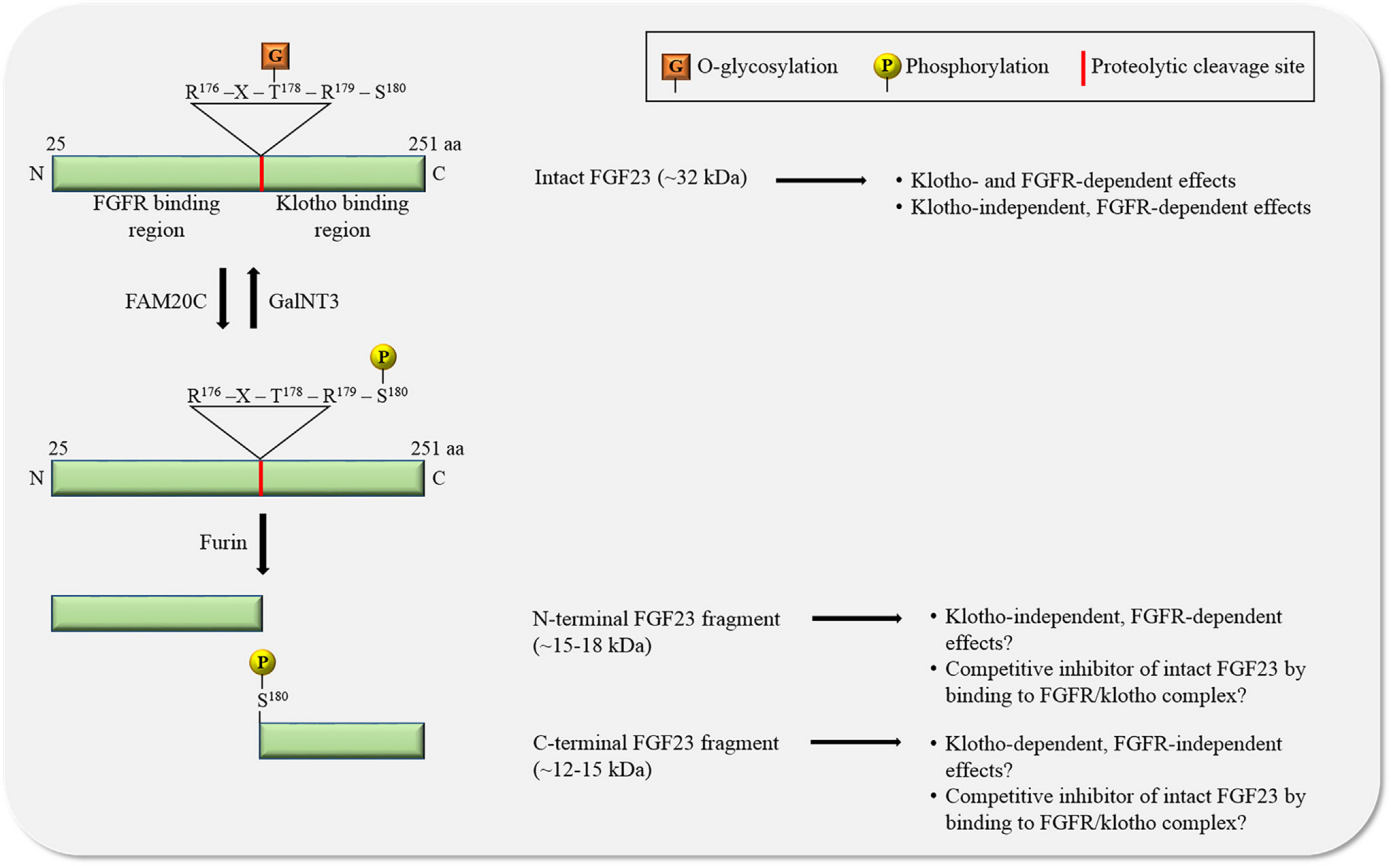
The regulation of FGF23 by posttranslational modifications. O-glycosylation by *N*-acetylgalactosaminyltransferase 3 (GalNT3) at threonine residue 178 (T^178^) protects FGF23 from proteolytic cleavage resulting in the generation of intact, biologically active FGF23. Phosphorylation of FGF23 on serine residue 180 (S^180^) by family with sequence similarity-20 member C (FAM20C) prevents O-glycosylation and promotes proteolytic cleavage by furin proteases. The biological relevance of the two generated FGF23 fragments is unclear. Abbreviations: aa, amino acid; N, N-terminus; C, C-terminus.

The biologically active form of FGF23 is a 32 kDa glycoprotein with a conserved N-terminus that shares homologies with the other FGF family members and contains a conserved FGF receptor (FGFR) binding site (18–20) (Figure 2). As the case for all endocrine FGFs, the C-terminus of FGF23 has only low binding affinity for heparin and instead is capable of interacting with alpha-klotho (termed klotho from here on) (20–24), a member of a family of three transmembrane proteins that act as FGFR co-receptors for endocrine FGFs (6, 25). The half-life of circulating FGF23 is about 45–60 min in humans (26), and appears to be much shorter in rodents, with about 20 min in mice (27) and 5 min in rats (28). Renal extraction seems to be a major contributor to FGF23 metabolism, while renal FGF23 excretion might play a minor role as FGF23 cannot be detected in urine, at least not in rodents (28). However, FGF23 can be measured in urine from patients with acute kidney injury (AKI), where elevations correlate with mortality (29). Whether in the context of AKI urinary FGF23 is derived from circulating filtered FGF23 or produced by the injured kidney is currently not clear.

## FGF23—Posttranslational Modifications and Processing

FGF23 can be cleaved by subtilisin-like pro-protein convertases, such as furin, at a consensus sequence (Arg^176^-X-X-Arg^179^) that is not present in other FGF family members (30–32) (Figure 2). FGF23 is O-glycosylated at several sites (31, 33, 34), and O-glycosylation at Thr^178^ by polypeptide *N*-acetylgalactosaminyltransferase 3 (GalNT3) protects FGF23 from proteolytic cleavage (35). FGF23 is also phosphorylated at several serine residues (36), and phosphorylation at Ser^180^ *via* the secretory protein kinase family with sequence similarity-20 member C (FAM20C), also called dentin matrix protein 4, inhibits GalNT3-mediated O-glycosylation, and thereby promotes proteolytic cleavage of FGF23 (37). A tight regulation of FGF23’s posttranslational modifications and processing is crucial, as mutations in modification sites and interference with processing can block or promote FGF23 cleavage, leading to elevated serum levels of intact FGF23 and hypophosphatemia (32, 38, 39) or to reduced serum levels of intact FGF23 and hyperphosphatemia (33, 34, 40–42), respectively, both associated with mineral bone disorders.

Furin-mediated cleavage of FGF23 results in the generation of two fragments and thereby separates the binding domains for FGFRs and klotho from each other (Figure 2). As FGF23 appears to act in concert with FGFR and klotho, it has been assumed that the two FGF23 fragments by themselves are inactive, as supported by injection studies in mice showing that both fragments lack phosphaturic activity (31). Interestingly, injections of the C-terminal fragment containing the klotho binding site in a genetic mouse model with high serum concentrations of FGF23, reduce FGF23 excess and associated renal phosphate wasting (22, 43), suggesting that FGF23 cleavage not only removes the klotho binding site from FGF23, but also generates an endogenous inhibitor of FGF23. While the mechanism underlying such an inhibitory action is not understood, it is plausible to speculate that C-terminal FGF23 can interact with klotho without binding and activating FGFRs, thereby competitively blocking access of intact FGF23 to the FGFR/klotho complex and inhibiting FGF23-induced signaling. The existence of such a mechanism is supported by an *in vitro* study, showing that the FGF23-mediated reduction of phosphate uptake in proximal tubular cells is blocked in the presence of the C-terminal FGF23 fragment (44). However, this view has been challenged by a different injection study in mice showing that C-terminal FGF23 retains phosphaturic activity (45), indicating that either the C-terminus by itself can bind FGFRs or that the fragment’s cellular actions are FGFR-independent and possibly mediated by other receptors. Furthermore, cell culture studies with chimeric FGF23:FGF21 proteins have shown that the replacement of the C-terminal klotho-binding site in FGF23 does not result in a loss of FGF23’s ability to activate FGFR/klotho-mediated signaling (46), suggesting that the N-terminus of FGF23 by itself can bind klotho. Nevertheless, a recent analysis of the crystal structure of the FGF23/FGFR1/klotho ternary complex clearly indicates that the N-terminus of FGF23 interacts with FGFRs, while FGF23’s C-terminus is bound to klotho (47).

It is currently unclear whether FGF23 cleavage fragments are biologically active, and if so, whether this activity differs from the actions of intact FGF23. One could speculate that furin-mediated cleavage serves as a first step in further proteolysis and removal of FGF23. However, since the same bone cell not only synthesizes, but also cleaves FGF23 and is, therefore, capable of releasing intact FGF23 as well as FGF23 fragments into the circulation (48), it is reasonable to assume that the fragments have a function and are not just a proteolytic garbage product. As FGF23 synthesis and cleavage appear to be independent events, they provide two distinct levels for the regulation of FGF23 production (48). It is possible that classic factors associated with mineral metabolism [such as phosphate, calcium 1,25D, and PTH (11, 49–52)] and novel factors linked to pathologic scenarios [such as systemic elevations of inflammatory cytokines, iron deficiency, and hypoxia (53–56)] regulate FGF23 production in osteocytes and osteoblasts at different levels. The massively elevated serum levels of intact FGF23, as observed in late stages of chronic kidney disease (CKD) (57, 58), seem to result from an increase in FGF23 synthesis (59) accompanied by an inhibition of FGF23 cleavage (53, 60). The inducers and mechanisms of FGF23 production and processing in bone are currently studied by many investigators [as reviewed in more detail elsewhere (48, 61–64)], and their characterization should provide important answers to one of the key questions in the field, i.e., why circulating FGF23 is elevated in CKD, as well as novel pharmacological targets to lower serum FGF23 levels. Furthermore, it needs to be determined whether cleavage of FGF23 only occurs in bone or also in the circulation and/or other tissues, which would suggest that the half-life of circulating FGF23 can be regulated and might be increased in CKD, and whether distant organs, such as the kidney, can control the production and processing of FGF23 in bone cells. Moreover, it is likely that changes in renal clearance of FGF23 might also contribute to CKD-associated FGF23 elevations.

## Klotho—A Protein that Comes in Multiple Forms

Klotho was originally identified as an anti-aging protein, because genetically modified mice lacking klotho develop a variety of phenotypic features that are associated with premature aging, including multiple organ dysfunction and a significantly shortened life span (65). The klotho gene encodes a 130 kDa single-pass transmembrane protein that is composed of two extracellular domains, termed KL1 and KL2, a transmembrane domain and a short cytoplasmic tail (66, 67). The KL1 and KL2 domains show amino acid sequence homologies with β-glucosidase of bacteria and plants, and, therefore, klotho could potentially catalyze the release of glucose from oligosaccharides (65). However, based on the absence of two conserved glutamic acid residues that are important for enzymatic activity of this family (65), it appears that klotho has only weak glucosidase activity, if any. A recent structural analysis of the klotho ectodomain combined with an *in vitro* assay to detect glycosidase, sialidase, and β-glucuronidase activities confirmed that klotho lacks enzymatic activity (47).

Klotho is mainly expressed in the kidney, brain, and parathyroid gland (65, 68, 69). In addition to the membrane-associated full-length protein, the ectodomain of klotho can also exist in a soluble form (soluble klotho, sKL), which can be generated by proteolytic cleavage of full-length klotho *via* the α-secretases, a desintegrin and metalloproteinase (ADAM) 10 and ADAM17, leading to sKL shedding from the cell membrane (70–73). sKL cleavage can also be mediated by the β-APP cleaving enzyme 1 (BACE1), which belongs to the family of β-secretases, and the remaining membrane-associated klotho fragment is further processed and removed by the γ-secretase complex (71, 73). The kidney is the major source for sKL (73–75), but also ependymal cells of the choroid plexus in the brain might release sKL by shedding (76–78), and sKL can be detected in the blood (73, 79–84) and the cerebrospinal fluid (CSF) (83, 84). The half-life of sKL in rats is about 7 h (73), which might be shortened in CKD, where degradation of circulating sKL appears to be increased (79). It seems that sKL is cleared from the circulation by the kidney, most likely by transport across renal tubules to the apical membrane and release into the urinary lumen (73), and sKL can be measured in the urine (73, 79–82). However, other studies have failed to detect sKL in urine (85), questioning if and how sKL can enter the urinary space.

## sKL—An Endocrine Factor with Pleiotropic Functions

It has been postulated that sKL acts as an endocrine factor that can target a variety of tissues (25, 86–88), but a specific receptor for sKL has not been identified to date, and the mechanisms underlying potential direct actions of sKL on target cells are only poorly understood. Several *in vitro* studies in multiple different cell types, such as fibroblasts, endothelial cells, vascular smooth muscle cells, cardiac myocytes, pulmonary epithelial cells, oligodendrocytes, and neurons, indicate that sKL has cell-protective activities, including the inhibition of apoptosis, oxidative stress, senescence, and pathologic gene programs (80, 89–107), suggesting that sKL might protect against cellular dysfunction as well as tissue fibrosis and inflammation. It has been postulated that such protective actions of sKL also exist *in vivo*, and that a reduction in circulating sKL levels, as observed in aging or in diseases, such as CKD, contribute to widespread tissue injury (86, 87, 108). However, strong experimental evidence indicating that sKL protects tissues by directly targeting them is still missing. Furthermore, it remains unclear if pathologies associated with a global reduction in klotho expression are caused directly by the absence of sKL and its tissue-protective actions, or indirectly by the loss of membrane-associated klotho resulting in systemic alterations, such as elevations in serum levels of phosphate or FGF23 (109). To distinguish experimentally between both scenarios, animal models with preserved expression of membrane-associated klotho, but a loss of sKL production [e.g., by genetically inactivating the proteolytic cleavage site in klotho (72)] need to be generated and analyzed. Since mice with kidney-specific deletion of klotho develop the same phenotype as mice that lack klotho globally (74), sKL derived from other sources than the kidney appears to lack tissue-protective effects and might not be capable of compensating for a loss of kidney-derived sKL.

To date the molecular base for the potentially pleiotropic actions of sKL remains a mystery. Several ligand/receptor complexes, such as insulin/IGF1/IGF1R (110–113), TGFβ1/type-II TGFβ receptor (114, 115), AngII/AT1R (96, 116), and Wnt/Frizzled (115, 117–119), have been postulated to serve as direct sKL targets (88). However, it is unclear how one particular protein can inhibit various signal mediators and receptors that significantly differ in their structure, biophysical features, and mode of action. Recently, a different mechanistic explanation has been suggested for sKL’s pleiotropic effects (87). The two KL domains of sKL can bind sialic acid, thereby targeting monogangliosides, such as GM1 and GM3, in cell membranes (120). Since the binding affinity of each KL domain for sialic acid is low (121), sKL preferentially interacts with lipid raft domains where gangliosides are enriched. The association with sKL might then affect overall lipid raft dynamics and composition, thereby regulating the localization and activity of a variety of raft-associated proteins, including signaling receptors and ion channels (87). This hypothesis is supported by *in vivo* findings showing that raft-associated, but not raft-independent, phosphoinositide 3-kinase (PI3K) signaling is elevated in mice lacking klotho (120). Furthermore, by binding to sialic acid on transmembrane proteins, sKL can regulate their cell surface abundance. This has been shown for transient receptor potential vanilloid type 5 (TRPV5), renal outer medullary potassium channel 1 (ROMK1) and NaPi-2a, which are all located in renal tubular cells (81, 122–124), suggesting that sKL might regulate ion homeostasis (125). It has been reported that sKL has sialidase and β-glucoronidase activity (81, 122–124, 126), indicating that sKL not only binds, but also removes sialic acid from lipids and proteins. However, the recent structural and functional analysis of sKL described earlier refutes such a hypothesis (47). Binding sialic acids in glycolipids and glycoproteins is a plausible mechanistic explanation for sKL’s pleiotropic actions (87). To further test this hypothesis, the respective binding site in sKL needs to be characterized and genetically inactivated followed by the functional characterization of the resulting sKL mutant in cell culture and animal models. Furthermore, since all eukaryotic cells contain lipids rafts (127), it is unclear how the described mechanism could ensure target specificity for sKL’s action.

Based on the low binding affinity of each KL domain for sialic acid, it has been suggested that sKL acts as a multimer (87). However, strong experimental data supporting the existence of sKL oligomers is still missing. Overexpression studies in cultured cells indicate that full-length klotho is capable of forming dimers (83, 128), and sKL might exist in an oligomeric form in serum and CSF from human and mice (83). Furthermore, klotho and sKL appear to be N-glycosylated (83, 129), but to date a detailed characterization of their posttranslational modifications including functional consequences has not been conducted. Interestingly, sKL might have intracellular activity, as suggested by a recent study (129). sKL can interact with a variety of cytosolic proteins, some of which are involved in regulating cellular anti-oxidative activities or posttranslational modifications and folding of other proteins. While this mechanism needs further experimental validation, including an explanation of how sKL secretion is blocked or bypassed, it is tempting to speculate that its dysregulation in klotho-expressing tissues might contribute to aging-related injury. A potential intracellular role of klotho is also supported by the fact that the klotho-related protein, KLrP, acts as a cytosolic enzyme (130).

## FGFR-Mediated Signal Transduction

The mammalian genome encodes four different FGFR isoforms (i.e., FGFR1-4) (1). FGFRs are receptor tyrosine kinases which are composed of an extracellular domain consisting of three immunoglobulin-like domains and containing the ligand-binding site, a transmembrane domain, and a cytoplasmic tyrosine kinase domain (131). Upon FGF binding, FGFRs form dimers and auto-phosphorylate each other at specific tyrosine residues within their cytoplasmic tails which then initiates subsequent downstream signaling events (132). The FGFR isoforms differ in their affinity for particular FGF ligands. Alternative splicing events increase the variety of FGFR1-3 isoforms, designated as b and c splice variants, thereby increasing the spectrum of FGFRs with distinct FGF binding specificities (1, 131). Although *in vivo* proof is still missing, *in vitro* studies suggest that FGFR isoforms not only form homo- but also heterodimers (133–135), which would further increase the possible combinations of FGFRs to form dimeric complexes with different FGF binding specificities.

FGFs ligands require the presence of co-receptors for efficient FGFR binding. For paracrine FGFs, this co-receptor is heparin/heparan sulfate which captures the FGF upon release and forms a stable complex with FGFRs in an isoform-dependent manner on the same or neighboring cell (131). Endocrine FGFs, such as FGF23, have significantly reduced affinity for heparin (21, 136). This feature enables FGF23 to avoid being captured by extracellular matrices and to function as a hormone, while it also reduces the capacity of heparin to promote FGF23 binding to FGFRs (137). Instead of heparin, klotho acts as an FGF23 co-receptor that promotes efficient binding of FGF23 to FGFRs (138, 139). Biochemical binding studies have shown that klotho increases the FGFR binding affinity of FGF23 by about 20-fold (23). The recent report of its atomic structure revealed that FGF23, sKL, and FGFR1c form a 1:1:1 complex, where sKL functions as a scaffold protein that brings FGFR and FGF23 in close proximity, thereby conferring stability of the ternary complex (47). Surprisingly, while heparan sulfate does not affect the formation and stabilization of the monomeric complex, it is required for complex dimerization, suggesting that the active signaling complex consists of FGF23, sKL, FGFR1, and heparan sulfate in a 2:2:2:2 stoichiometry (47). While FGFRs are widely expressed, the restricted expression of klotho to renal tubules and parathyroid gland defines FGF23 target tissues (15, 65, 75). It is plausible to assume that besides heparin and klotho, many other co-factors participate in the formation of FGF/FGFR signaling complexes, such as cell adhesion proteins of the cadherin and immunoglobulin superfamilies (140–145), and modify the binding affinity, accessibility, and activity of FGFRs.

Fibroblast growth factor receptor signaling is transduced by the cytoplasmic adaptors, phospholipase Cγ (PLCγ), and FGF receptor substrate 2α (FRS2α) (1, 132). Following ligand-induced auto-phosphorylation of FGFR, PLCγ binds directly to one specific phosphorylated tyrosine residue within the FGFR cytoplasmic tail (146, 147). Subsequent tyrosine phosphorylation of PLCγ results in PLCγ activation by the receptor (148). Downstream signal transduction is mediated by PLCγ-catalyzed production of diacylglycerol and inositol 1,4,5-triphosphate that can increase cytoplasmic calcium levels thereby inducing the activation of several calcium-sensing signal mediators, including the protein phosphatase calcineurin (131). The dephosphorylation of the transcription factor, nuclear factor of activated T cells (NFAT), by activated calcineurin causes the translocation of NFAT into the nucleus to modulate the expression of specific target genes (149). FGFR signaling can also be transduced *via* the activation of FRS2α by FGFR-mediated tyrosine phosphorylation (131). In contrast to PLCγ, FRS2α is constitutively bound to FGFR independently of the receptor’s activation state (150). FRS2α-mediated signaling results in the activation of Ras/mitogen-activated protein kinase (MAPK) and PI3K/Akt signaling (131).

**Figure 3:**
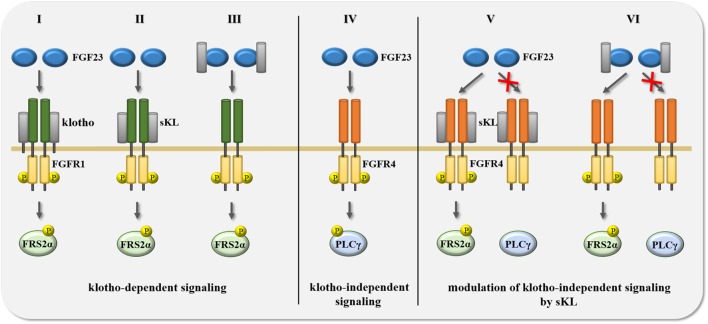
Summary of potential scenarios for the crosstalk between FGF23 and klotho in the regulation of signal transduction. FGF23-activated signaling events are mediated and modified by the availability of either membrane-associated full-length klotho or soluble klotho (sKL). (I–III) Transmembrane klotho as well as sKL mediate FGF23 binding to FGFR1 resulting in the phosphorylation of FGF receptor substrate 2α (FRS2α) and the subsequent activation of Ras/mitogen-activated protein kinase (MAPK) signaling. (IV) In the absence of klotho and sKL, FGF23 binds FGFR4 which induces the phosphorylation of phospholipase Cγ (PLCγ) and the activation of calcium-regulated signal pathways, such as calcineurin/NFAT. (V–VI) Membrane-associated klotho and sKL can modulate klotho-independent FGF23/FGFR4 signaling in two different ways. First, in the presence of klotho or sKL, FGF23 cannot bind to FGFR4, and thereby not activate the PLCγ-driven signaling cascade. Second, binding of FGF23 to FGFR4 can also occur in the presence of transmembrane klotho or sKL, but causes different downstream events, switching from PLCγ to FRS2α phosphorylation, thereby activating Ras/MAPK instead of calcineurin/NFAT signaling. Abbreviation: P, protein phosphorylation.

Although FGFRs can stimulate a variety of downstream signaling branches (132), most FGF and FGFR isoforms have been shown to employ the FRS2α/Ras/MAPK pathway to mediate their cellular effects. This is also the case for FGF23. In the presence of klotho, FGF23 can bind to the c splice variants of FGFR1-3 as well as to FGFR4 which results in the activation of MAPK signaling (47, 138, 139). In FGF23’s physiologic target organs (15, 16, 151–154), FGFR1 appears to be the main FGF23 receptor which acts in concert with klotho (Figure 3, I). It has been shown in HEK293 cells, which express different FGFR isoforms, including FGFR1, but lack klotho, that the forced overexpression of full-length klotho followed by FGF23 treatment results in FRS2α/Ras/MAPK signaling (47, 128, 155, 156). Interestingly, the same effect was observed when HEK293 cells were co-treated with FGF23 and sKL (47, 139). These *in vitro* studies show that the introduction of klotho is sufficient to make FGFR expressing cells responsive to FGF23 resulting in an activation of FRS2α/Ras/MAPK signaling. Furthermore, they indicate that membrane-associated klotho and sKL share a common function, which is that both mediate FGF23-induced FRS2α/Ras/MAPK signaling.

## FGF23 and Klotho Co-Regulate FGFR-Mediated Signaling

Overall, one could speculate that sKL acts as a circulating FGF23 co-receptor that promotes an interaction between FGF23 and membrane-bound FGFRs, thereby facilitating FGF23 binding to cell types that *per se* do not express klotho and mediating temporary responsiveness to FGF23. Such a mechanism has been reported in fibroblast and myoblast cell lines in which the combined treatment with FGF23 and sKL activates FGFR1/MAPK signaling leading to increased cell survival (157). Furthermore, combined treatment with FGF23 and sKL induces Ras/MAPK signaling in cultured osteoblasts, which is FGFR1-dependent and results in increased FGF23 production (158). FGF23 and sKL together also induce the phosphorylation of FRS2α and MAPK in cultured chondrocytes, which seems to be mediated by FGFR3 (159). Interestingly, elevating serum sKL levels in mice by viral overexpression of sKL in the liver or by injection of recombinant sKL protein causes increased Ras/MAPK signaling and reduces NaPi-2a expression in the kidney resulting in increased renal phosphate excretion and hypophosphatemia (47, 157). When conducted in mouse models with CKD or genetic klotho deficiency, such sKL elevations reduce renal NaPi-2a expression as well as the increases in serum phosphate levels (158). Combined, these animal studies suggest that sKL can compensate for the reduction or loss of klotho, and that by targeting the kidney, sKL might increase FGF23-mediated FGFR1/Ras/MAPK signaling in tubular cells, thereby reducing renal phosphate uptake and lowering serum phosphate levels. One could speculate that sKL acts as a circulating factor that elevates FGF23 production in the bone and mediates FGF23 signaling in target organs by promoting FGFR1 binding (160). This view is supported by the recent finding that sKL lacking the FGFR binding domain does not show phosphaturic activity when injected into mice and cannot mediate FGF23-induced Ras/MAPK signaling in HEK293 cells. Similarly, mutant forms of sKL and FGF23 which disrupt sKL-FGF23 binding fail to activate Ras/MAPK signaling (47).

As described before, a recent structural analysis shows that sKL acts as a scaffold protein for FGF23 and FGFRs that promotes FGF23/FGFR-mediated signaling, suggesting that all effects of sKL are FGF23-dependent and thereby challenging the concept that sKL can function as an FGF23-independent hormone (47). A crystal structure can only provide a snap shot of one particular state within a multi-step signaling process and the precise order of binding events among the signal inducers remains unknown. Two scenarios seem to be possible. First, sKL binds a membrane-associated FGFR which then enables FGF23 binding to the receptor complex (Figure 3, II). It has been shown that sKL has high affinity for FGFR1c (22), and experiments in cell culture overexpression systems show that full-length klotho can bind FGFRs in the absence of FGF23 (139), suggesting that sKL might have similar abilities. It remains to be established whether endogenous sKL binding and effects are FGFR isoform-specific. As mentioned before, studies in HEK293 cells with overexpressed full-length klotho or with sKL incubations suggest that klotho preferably binds to FGFR1c, FGFR2c, FGFR3c, and FGFR4 (47, 139). Second, FGF23 and sKL bind to each other in solution and then together interact with an FGFR on the cell surface (Figure 3, III). Although the sKL/FGF23 complex has been detected in the circulation of rodents (161) and in extracts and supernatants from transfected HEK293 cells (159), surface plasmon resonance spectroscopy studies have shown that sKL binds FGF23 only with very low affinity in the absence of FGFR (22). Clearly, more experimental work is required in order to determine whether FGF23 and sKL can interact with each other in solutions, such as cell culture supernatants or blood.

Studies in HEK293 cells and in primary cardiac myocytes showed for the first time that FGF23 can also affect cell types that do not express klotho (162), implying that the presence of klotho is not a prerequisite for FGF23 responsiveness. Whereas klotho-expressing cells respond to FGF23 by activating the FRS2α/Ras/MAPK cascade (138), FGF23 stimulates PLCγ/calcineurin/NFAT in cells that lack klotho (155, 162, 163). Furthermore, while FGFR1 acts as the main FGF23 receptor in “classic” klotho-expressing FGF23 target organs (138, 139), klotho-independent effects of FGF23 appear to be mediated by FGFR4 (Figure 3, IV) (155, 163, 164). In HEK293 cells, FGF23 only induces PLCγ binding to FGFR4, but to none of the other FGFR isoforms (155). Furthermore, deletion of FGFR4 or co-treatment with FGFR4-specific blocking agents inhibits FGF23-induced PLCγ/calcineurin/NFAT signaling in cardiac myocytes (155). Finally, FGF23 can activate the PLCγ/calcineurin/NFAT pathway in cultured hepatocytes, which of all FGFR isoforms only express FGFR4 (163). Overall, it appears that depending on the FGFR isoform and the presence or absence of klotho, FGF23 activates distinct downstream signaling pathways. The FGFR4/PLCγ/calcineurin/NFAT cascade seems to be a major mediator of klotho-independent FGF23 signaling (109), whose tissue-specific effects are discussed in detail below.

Since elevations in serum FGF23 levels, as observed in aging and in CKD, correlate with decreases in renal expression of klotho and in circulating levels of sKL (80, 165), it is interesting to speculate that associated pathologies are caused by both, high FGF23 and low sKL. If true, one would assume that sKL might act as an inhibitor of klotho-independent pathologic actions of FGF23. The existence of such a mechanism is supported by a study in vascular smooth muscle cells and endothelial cells, where sKL counterbalances the effects of FGF23 (98). As discussed earlier for the stimulating effects of sKL on FGF23/FGFR1 signaling, it is possible that sKL inhibits FGF23/FGFR4 signaling by either binding first to FGFR4 (Figure 3, V), or *via* an initial interaction with FGF23 (Figure 3, VI). sKL could then block the interaction between FGF23 and FGFR4 and thereby inhibit PLCγ/calcineurin/NFAT signaling. It is possible that sKL acts as a circulating FGF23 decoy receptor, as described for other ligands and truncated forms of their transmembrane receptors, such as vascular endothelial growth factor (VEGF) and the soluble form of VEGF receptor-1 (called sFlt-1) generated by alternative splicing (166). It is also possible that sKL can bind FGFR4 and block the access or affinity of FGFR4 for FGF23. A similar mechanism has been reported in cultured proximal tubular epithelial cells, where sKL binding to FGFR1 inhibits FGF2/FGFR1 signaling, most likely by competing with FGF2 for FGFR1 binding (101). Such a mechanism might also underlie sKL’s inhibitory actions toward FGF2-induced Ras/MAPK signaling and proliferation in tumor cells (112). As an alternative mechanism, FGFR4 and FGF23 might still be able to form a complex in the presence of sKL, but activate FRS2α/Ras/MAPK rather than PLCγ/calcineurin/NFAT downstream signaling, resulting in significant differences in cellular effects (Figure 3, VI).

Shedded sKL consists of the KL1 and KL2 domains (70–73). Furthermore, it has been reported that also the KL1 domain alone can be generated by proteolytic cleavage from membrane-associated klotho (70) or by alternative splicing and subsequent secretion into the extracellular environment (66, 67). However, a more recent study indicates that alternative splicing is not involved in the generation of any soluble forms of klotho, as alternative klotho mRNA transcripts are primed for nonsense-mediated mRNA decay and are not translated into protein (167). Whether soluble KL1 actually exists in animals and in humans, and if so, is present in the circulation and can act as a hormone, needs to be established. Since the KL1 domain seems to be sufficient for sialoganglioside binding (121), and KL1 has tumor suppressor activity *in vitro* and *in vivo* (112, 156), one could speculate that KL1 by itself has biological activity. However, studies with recombinant proteins have shown that KL1 cannot mediate FGF23-induced Ras/MAPK signaling in cultured cells (156), and when injected into mice, KL1 does not lower serum phosphate levels (112). A recent structural study reporting that sKL binding to FGF23 and FGFR1 is mediated by KL2 and the linker region connecting KL2 with KL1 supports the idea that KL1 by itself might not have biological activity, at least no activity that requires FGF23 and FGFRs (47).

## Effects of FGF23 on the Heart

The heart was the first organ that was shown to respond to circulating FGF23 in a klotho-independent fashion (162). FGF23 directly targets cardiac myocytes *via* the described FGFR4/PLCγ/calcineurin/NFAT signaling pathway and induces cardiac hypertrophy, and potentially other changes in cardiac remodeling, including cardiac fibrosis and altered cardiac metabolism, eventually resulting in reduced heart function (155, 162, 164) (Figure 1). Animal models with elevated serum FGF23 levels, induced by CKD, genetic deletion of klotho, injection of recombinant FGF23 protein, or a high phosphate diet, develop cardiac hypertrophy, which can be blocked by administration of specific inhibitors for FGFR4 or calcineurin (155, 164, 168). Since only some of these animal models develop kidney injury, hyperphosphatemia, or hypertension, while all of them have elevated FGF23 and cardiac hypertrophy, one can assume that by directly targeting the myocardium, FGF23 is a major driver of cardiac remodeling that acts independently of other pro-hypertrophic factors, such as high blood pressure or uremic toxins. However, these animal models also show low serum sKL levels, and it seems that in all scenarios studied to date, FGF23 elevations are always accompanied by a decrease in sKL concentrations (109). Since animal experiments indicate that sKL reductions *per se* can contribute to cardiac injury (80, 107, 165, 169), it would be important to conduct studies in the absence of FGF23 in order to determine whether in the context of CKD, FGF23 elevations are required for the induction of cardiac injury. However, such loss-of-function studies are not feasible, as FGF23 deletion or inhibition in rodents elevates serum phosphate levels and causes severe cardiovascular injury resulting in premature death (12, 170). Instead, the cardiac myocyte-specific deletion of the FGF23 receptor, i.e., FGFR4, followed by the elevation of circulating FGF23 should help to determine whether direct cardiac actions of FGF23/FGFR4 contribute to cardiac hypertrophy.

Several studies have shown that repetitive intravenous and intraperitoneal injections of recombinant FGF23 protein in wild-type mice induce cardiac hypertrophy within 5 days, indicating that FGF23 has potent hypertrophic effects on the heart (162, 171–173). However, it remains unknown whether FGF23 elevations are also sufficient to impair cardiac function and cause heart failure. Future experiments in animal models with increased serum FGF23 levels need to study the observed changes in cardiac structure and function in more detail, and put them into context with particular FGF23 exposure concentrations and times, which is experimentally challenging. It has been hypothesized that long-term exposure at very high FGF23 concentrations, as the case in patients with late stages of CKD, who can develop up to 1,000-fold elevations for months (174, 175), causes pathological cardiac remodeling and contributes to uremic cardiomyopathy (109, 176–178). Such a hypothesis is supported by a recent study in mice lacking the alpha 3 chain of type IV collagen (Col4a3), a genetic animal model for CKD (179). Dependent on the genetic background, these mice either develop fast-progressing kidney injury and die at around 10 weeks of age, or the development of severe kidney injury takes longer resulting in extended survival until about 20 weeks (180, 181). Although both mouse lines show the same degree in blood pressure elevations, only slow-progressing Col4a3 knockout mice develop cardiac hypertrophy and fibrosis which is accompanied by increased cardiac expression levels of FGFR4 (179). At 10 weeks of age, fast-progressing Col4a3 knockout mice show significantly higher elevations in intact FGF23 concentrations than slow progressors. Although serum FGF23 levels in slow-progressing mice further increase between 10 and 20 weeks, they never reach the levels of those detected in fast progressors. Overall, this study indicates that the duration of the exposure to increased circulating FGF23, rather than the degree of FGF23 elevation *per se*, is an important determinant in the development of pathologic cardiac remodeling in this model of CKD. Future studies should determine the impact of cardiac FGF23/FGFR4 signaling in comparison to other potential factors, such as hyperphosphatemia or hypertension, which based on the increased duration of impact might also have more damaging effects in slow-progressing Col4a3 knockout mice. Furthermore, detailed cardiac analyses of the rodent model for adenine-induced tubulointerstitial nephropathy, where serum FGF23 levels are highly elevated (182) and appear to positively correlate with cardiac hypertrophy (183), should help to further determine a causative role of FGF23 in uremic cardiomyopathy.

Elevations in cardiac FGFR4 expression and calcineurin activity have not only been reported in animal models of CKD (155, 168, 184), but also in patients with CKD (185). A retrospective study with autopsy samples from heart tissue of 24 deceased pediatric patients showed that only individuals who had developed cardiac hypertrophy also showed significant elevations of FGFR4 in the heart, but not of FGFR1, as well as of calcineurin and NFAT (185). In these patients, FGFR4 expression positively correlated with the size of individual cardiac myocytes suggesting a causative relationship between FGFR4/PLCγ/calcineurin/NFAT activation and the induction of cardiac hypertrophy in humans. This is further supported by the finding that compared to dialysis patients who remained high FGF23 concentrations, patients who received a kidney transplant and had reduced FGF23 levels also showed lower cardiac expression of FGFR4, calcineurin, and NFAT (185). In the same study, CKD patients also showed the presence of sKL protein in the heart which must be derived from the circulation, as no increase in cardiac klotho mRNA could be detected. Interestingly, in this study declining sKL protein levels in the heart in combination with elevated FGF23 production correlated with cardiac hypertrophy, indicating that a reduction in sKL’s inhibitory actions toward FGF23’s pro-hypertrophic effects might contribute to uremic cardiomyopathy (185), thereby supporting the hypothesis that sKL can act as a decoy receptor for FGF23, as discussed above.

Several cell culture studies indicate that cardiac myocytes serve as a direct target for FGF23 (155, 162, 164, 186–188). Besides the activation of pro-hypertrophic gene programs (162, 188), FGF23 might also induce the expression of pro-fibrotic factors and inflammatory cytokines in cardiac myocytes, thereby contributing to cardiac injury (188). Furthermore, it has been recently shown that FGF23 can activate cardiac fibroblasts isolated from adult mice and newborn rats (188, 189), and that the experimental elevation of FGF23 expression in an animal model for myocardial infarct further increases cardiac fibrosis (189). Therefore, it is possible that by directly targeting cardiac fibroblasts, FGF23 contributes to cardiac fibrosis and pathologic cardiac remodeling (Figure 1). The underlying mechanism is not clear, but since cardiac cells, including fibroblasts, in humans and rodents do not express klotho (75, 162, 184, 185), it would be worth to study a potential involvement of the FGFR4/PLCγ/calcineurin/NFAT signaling cascade. It has been recently shown that fibroblasts isolated from an injured mouse kidney can respond to FGF23 resulting in the activation of pro-fibrotic gene programs (190), as discussed below. Interestingly, this FGF23 effect on injury-primed renal fibroblasts was mediated by FGFR4 and PLCγ/calcineurin/NFAT signaling and resulted in the activation of the pro-fibrotic transforming growth factor β (TGFβ) signaling cascade (191). Based on these studies, one could hypothesize that FGF23 responsiveness is also increased in injured cardiac fibroblasts. It would be interesting to determine effects of FGF23 on cardiac fibroblasts isolated from uremic hearts. Although they do not provide evidence for direct and causative actions of FGF23 on the heart, some animal studies have indicated that FGF23 might require a fibrotic (189) or inflammatory milieu (192, 193) in order to contribute to cardiac injury. As suggested for the kidney (190, 191), FGF23’s pro-fibrotic effect on the heart might be also mediated by FGF23-induced TGFβ signaling (189). However, a recent study has reported that FGF23 does not elevate TGFβ in isolated neonatal rat cardiac myocytes or fibroblasts, and that other mediators, such as the renin–angiotensin–aldosterone system, connective tissue growth factor, or endothelin-1, might be involved in FGF23-induced alterations in the myocyte-fibroblast crosstalk that may contribute to cardiac fibrosis (188).

The concept of priming cardiac cells (and possibly of other cell types in the body) by tissue injury resulting in increased FGF23 sensitivity appears to be plausible. Although cause and mechanism of the priming event are not known, it is possible that CKD-related stressors, such as elevations in serum levels of phosphate or uremic toxins, increase the cell surface expression of FGFR4 which then senses FGF23 elevations. Since fibroblasts do not express FGFR4, or only at very low levels (155), this mechanism would fit with the concept of injury-induced FGF23 responsiveness of fibroblasts resulting in pro-fibrotic actions of FGF23. The hypothesis that FGF23 mainly contributes to cardiac injury in the context of CKD is also supported by two other findings. First, although serum FGF23 levels are elevated following pressure overload *via* transaortic constriction in mice, FGF23 does not appear to be required for the development of pathologic cardiac hypertrophy in this animal model (171). Second, patients with X-linked hypophosphatemia (XLH), genetic forms of hypophosphatemic rickets, have high serum levels of FGF23 but do not develop cardiac hypertrophy (194). However, another clinical study challenges this view (195). Furthermore, it has been shown that genetic mouse models for XLH and autosomal recessive hypophosphatemic rickets (ARHR) show an increase in cardiac mass, while cardiac functions seems to be normal (172, 173, 196). Overall, primary forms of pathologic cardiac remodeling might not require FGF23, and not every scenario of FGF23 elevations might result in cardiac hypertrophy. In the context of secondary CKD-associated cardiac damage, the type and severity of kidney injury might also affect FGF23’s actions on the heart. A recent study in a genetic mouse model with primary podocyte injury leading to a CKD-mineral bone disease-like phenotype shows that although serum levels of phosphate and FGF23 were elevated, mice showed no signs of pathologic cardiac remodeling (197). It is possible that the absence of a cardiac phenotype was based on the relatively short exposure time, as mice died within 8–10 weeks. Of note, although mice developed significant kidney injury, they did not have renal fibrosis. Therefore, one could speculate that mice did not develop cardiac hypertrophy, since they lack pathologic stimuli released by activated renal fibroblasts and/or associated with kidney fibrosis that act in synergy with FGF23 to harm the heart.

Cell culture and animals studies have shown that FGF23/FGFR4-induced hypertrophy is reversible upon removal or inhibition of the FGF23 stimulus (164). Furthermore, studies in a rat model of CKD have shown that administration of a FGFR4-specific blocking antibody (anti-FGFR4) not only prevents the induction of cardiac hypertrophy (155), but also blocks the progression of cardiac injury in animals with already established cardiac hypertrophy (164). Therefore, FGF23/FGFR4 might serve as a pathomechanism of uremic cardiomyopathy that could be tackled pharmacologically. Since FGF23 mediates its physiologic functions mainly *via* FGFR1, while its klotho-independent actions on the heart and other organs seem to be mediated by FGFR4 (155, 163, 191), a therapeutic approach to block FGFR4 should only interfere with FGF23’s pathologic actions, while leaving its role as an important regulator of phosphate metabolism unaffected. Since global FGFR4 knockout mice are viable and do not develop any significant phenotypic alterations (198), and delivery of anti-FGFR4 does not show toxic effects in rats (155), FGFR4 appears to be an appropriate drug target and its systemic blockade might only result in minor side effects. However, other findings dampen the excitement for FGFR4 blocking therapies. First, although anti-FGFR4 treatment of CKD rats as well as lowering elevated FGF23 levels by taking mice off a high phosphate diet, reduce cardiac hypertrophy, these interventions seem to have little or no effect on cardiac fibrosis (164). Compared to CKD patients who received a kidney transplant, dialysis patients have higher serum FGF23 levels and develop cardiac fibrosis, but cardiac FGFR4 expression levels do not correlate with fibrosis (188), further supporting the notion that cardiac FGF23/FGFR4 signaling might not directly contribute to fibrosis. If cardiac fibrosis persists following FGFR4 blockade, one would assume that although hypertrophy is halted, cardiac injury will progress and heart function will further decline, eventually resulting in heart failure. Clearly, future *in vitro* and *in vivo* studies need to determine cardiac effects of FGF23 in a cell type-specific manner to elucidate precise cardiac actions of FGF23 and its potential for drug development. Second, it has been shown that FGF23 also has acute effects on the myocardium. Within seconds, FGF23 increases cytoplasmic calcium levels in an FGFR-dependent manner in cultured cardiac myocytes (186). Furthermore, within minutes, FGF23 elevates the contractile force of isolated murine ventricular muscle strips, which does not occur in the presence of an FGFR4 inhibitor (164). Therefore, FGF23/FGFR4 might have beneficial effects on the heart which involve an increase in cardiac function, and its blockade might result in adverse outcomes. Whether acute effects of FGF23 on calcium homeostasis and contractility are indeed beneficial, or result in arrhythmia, as suggested by a different study (187), requires further analyses. Furthermore, it would be interesting to determine a potential involvement of FGF23 in the development of physiological hypertrophy, as observed during pregnancy and in athletes (199). However, to date it is still unknown whether under these two conditions of extreme physiologic and metabolic alterations, serum FGF23 levels are even elevated. Only one study has reported an increase of circulating FGF23 by about twofold in pregnant mice (200), and a different study showed that in professional cyclists, serum FGF23 levels increase by about 50% during a 3-week race (201). An elevation of FGF23 in these scenarios appears to be plausible, as they should both involve significant changes in calcium/phosphate homeostasis. However, analyses of larger populations are required to draw meaningful conclusions. Furthermore, to determine whether circulating FGF23 might contribute to physiologic cardiac hypertrophy requires studies in animals with genetic modifications of the FGF23/FGFR4 signaling pathway in the heart.

It appears that FGF23 can hit the heart in several ways: directly, by targeting cells in the myocardium, and indirectly, by contributing to traditional as well as non-traditional or CKD-specific cardiovascular risk factors (63, 125, 202). It has been shown that by targeting kidney distal tubules *via* FGFR1 and klotho, FGF23 elevates the expression of the sodium chloride co-transporter (NCC) and reduces levels of angiotensin-converting enzyme 2 (172, 173). By doing so, FGF23 increases sodium retention and activates the renin–angiotensin system, respectively. Combined, these renal effects lead to hypertension which is an established inducer of pathologic cardiac remodeling (199). FGF23 has been shown to be also involved in the regulation of iron metabolism by affecting erythropoiesis (62, 203). FGF23 reduces renal production of erythropoietin and might thereby contribute to CKD-associated anemia (204). Furthermore, FGF23 promotes expression of inflammatory cytokines in the liver (as discussed below) (163), and possibly in other tissues. Since *vice versa*, iron deficiency and inflammatory cytokines increase FGF23 production in bone (53), FGF23 could be part of a vicious cycle that contributes to FGF23-driven pathologies associated with CKD, such as anemia and systemic inflammation (62, 203), which are also potent inducers of pathologic cardiac remodeling and injury (199).

## Effects of FGF23 on the Liver

In mammals, the liver is among the organs with highest FGFR4 expression levels (205), raising the question if FGF23 has direct hepatic actions. Indeed, it has been shown that FGF23 can activate FGFR4/PLCγ/calcineurin/NFAT signaling in cultured hepatocytes which lack klotho, and thereby induce the expression of the inflammatory cytokines, interleukin-6 (IL-6) and C-reactive protein (CRP) (163). As already described for the associated cardiac remodeling, animal models with elevated serum FGF23 levels, induced by renal ablation, deletion of klotho, injection of FGF23, or administration of a high phosphate diet, show increased hepatic and serum levels of IL-6 and CRP, which are reduced when FGFR4 is deleted or pharmacologically inhibited (163). As discussed for the heart, hepatocyte-specific deletion of FGFR4 will be necessary to determine whether direct actions of the FGF23/FGFR4 signaling system on the liver contribute to systemic inflammation, and thereby to inflammatory injury in different tissues. Since studies in CKD patients have shown that higher serum FGF23 levels are associated with increased circulating concentrations of inflammatory cytokines, such as CRP, IL-6, IL-12, and tumor necrosis factor α (TNFα) (206–209), and that an elevation in these cytokines is a strong predictor of poor clinical outcome (210–213), the direct hepatic actions of FGF23/FGFR4 might serve as a novel pathomechanism that links CKD with systemic inflammation and contributes to morbidity and mortality (Figure 1). If hepatocytes and/or other cell types of the liver directly respond to FGF23 needs to be further investigated. Primary hepatocyte cultures are not absolutely pure and contain other cell types, such as Kupffer cells, which are specialized macrophages that release cytokines, such as IL-6 and TNFα, to communicate with hepatocytes (214). Since it has been shown that FGF23 can directly target other macrophage populations (215, 216), it is possible that FGF23 induces IL-6 production in Kupffer cells, which then indirectly affects hepatocytes.

In the described animal models with FGF23 excess, elevations in the hepatic production of inflammatory cytokines occur in the absence of increased liver enzymes (163). To date, no cell culture or animal studies have reported damaging actions of FGF23 on liver cells, which is consistent with the clinical observation that the presence of CKD *per se* does not promote liver injury despite marked increases in serum FGF23 levels. As discussed for cardiac hypertrophy, it is possible that FGF23-induced expression of inflammatory cytokines is not pathological, at least not initially, but has protective functions. Kupffer cell-derived IL-6 is a major regulator of hepatocyte proliferation and survival and thereby promotes liver regeneration (214). The hypothesis that FGF23/FGFR4 might act as a hepato-protective signaling pathway is supported by the finding that mice lacking FGFR4 are more sensitive to carbon tetrachloride-induced liver injury (217) and fail to restore liver mass after partial hepatectomy (218). Other FGF family members, such as paracrine FGF7 and FGF9, have been shown to mediate repair in response to liver injury (219). Further research is needed to determine whether physiologic concentrations or only high levels of FGF23, as observed in advanced CKD, can stimulate hepatic cytokine expression, and whether FGF23-induced cytokines have physiologic functions or mediate global tissue injury.

It is likely that FGF23 can stimulate inflammatory cytokine expression and secretion from other known reservoirs. This is supported by a genome-wide analysis of FGF23-regulated genes in a mouse model of CKD that suggested inflammatory cytokine genes as general FGF23 targets (220), and by studies in which FGF23 stimulated TNFα expression in macrophages (215, 216) and in the spleen (221). Furthermore, NFAT activation induces the expression of a variety of cytokines, such as IL-2, IL-4, IL-6, and TNFα, in different cells types, including T cells and mast cells (222). Therefore, it is possible that by activating klotho-independent calcineurin/NFAT signaling in other cell types, FGF23 induces the production of inflammatory cytokines in multiple tissues contributing to systemic elevations of inflammatory mediators as well as tissue injury (223).

Several factors that are dysregulated in CKD associate with elevations in inflammatory cytokines and might contribute to inflammatory tissue damage, including increased serum phosphate levels (224, 225). However, since only some of the studied animal models with elevated FGF23 develop kidney injury or hyperphosphatemia, while all of them show increases in serum concentrations of inflammatory cytokines (163), it is possible that FGF23 acts as a major driver of inflammation in CKD. Associations between serum levels of FGF23 and inflammatory cytokines have been also reported in adults without CKD and in the elderly, despite their significantly lower FGF23 levels relative to patients with CKD (226–228). This suggests a general pro-inflammatory role of FGF23 that is independent of reduced kidney function, and that FGFR4 blockade might be effective in reducing chronic inflammation. In CKD, several liver-controlled mechanisms, such as iron and lipid metabolism or detoxification, are out of order. Whether FGF23-mediated activation of FGFR4 in hepatocytes can also regulate these facets of liver function needs to be investigated. Klotho is not expressed in the liver (75, 163), and to date direct effects of sKL on the liver have not been reported. However, since a reduction in global klotho expression in rodents (163, 221, 229, 230) and humans (209) has been associated with systemic elevations of inflammatory cytokines, it is tempting to speculate that sKL’s anti-inflammatory effects are—at least partially—due to its inhibitory actions toward the FGF23-mediated production of inflammatory cytokines. While such a mechanism has been described in cultured endothelial cells where FGF23-induced expression of IL-1 is inhibited by sKL (231), it was not found in cultured spleen cells, where the FGF23-induced production of TNFα is not reduced in the presence of sKL (221).

## Effects of FGF23 on Leukocytes

Chronic kidney disease is a state of acquired immune deficiency involving cellular and humoral immunity (232). The incidence of bacterial infections in CKD patients is higher than in the general population, and acute infections with bacteria, viruses, and fungi substantially contribute to the high hospitalization rates and mortality (233–235). As described above, the pro-inflammatory CKD environment is most likely caused by a variety of sources, including activated immune cells, and *vice versa* systemic inflammation is associated with an impaired function of the immune system. Since in CKD patients elevated serum FGF23 levels are independently associated with the incidence of infections (175, 236, 237), a role of FGF23 not only in the regulation of the inflammatory response, but also in the associated host defense is plausible (223).

The pathomechanism underlying the impaired host defense in CKD is not well understood. The innate immune response requires the recruitment and activation of immune cells to the site of infection, and neutrophils are among the most prominent leukocyte subsets during this process. Neutrophils from CKD patients are unresponsive to further stimulation and activation, indicating that CKD-associated factors might contribute to defects in the host defense by inducing neutrophil dysfunction (238–240). A recent experimental study has shown that by directly targeting neutrophils, FGF23 can inhibit neutrophil recruitment and thereby impair the host defense (241) (Figure 1). Mechanistically, FGF23 blocks chemokine- and selectin-mediated β_2_-integrin activation on neutrophils, thereby preventing the interaction between β_2_-integrin and intracellular adhesion molecule-1 on endothelial cells, neutrophil arrest on the endothelium and trans-endothelial neutrophil migration. This FGF23 effect appears to be klotho-independent and requires FGFR activity. Since neutrophils only express FGFR2 on the cell surface, while FGFR1 and FGFR4 are localized in the cytoplasm (242), one can assume that FGF23 targets neutrophils *via* FGFR2. FGF23 actions on neutrophils seem to be dose-dependent and only occur at high FGF23 concentrations, which result in the activation of protein kinase A (PKA), subsequent inhibition of the signal mediator Rap1, and eventually deactivation of β_2_-integrin. This pathomechanism can be translated into humans, as leukocytes isolated from CKD patients show increased rolling velocity which can be reduced by pharmacologic FGFR inhibition, and *vice versa*, FGF23 treatment of leukocytes isolated from healthy subjects elevates rolling velocity (241). Furthermore, two more recent studies show that FGF23 treatment of isolated human leukocytes reduces expression levels of CD11b integrin (208) and weakens chemotaxis (243). Combined, these findings suggest direct inhibitory actions of FGF23 on integrin activation in leukocytes, resulting in reduced leukocyte adhesion and migration which might serve as a pathomechanism that causatively links increase in serum FGF23 levels with impaired host defense in CKD. Since integrin inactivation occurs rapidly following FGF23 treatment (241), this effect appears to be direct. However, the analysis of rodent models with cell type-specific deletion of FGFR2 will be required to determine whether FGF23 can indeed directly target neutrophils.

Direct effects of FGF23 have been also described for other leukocyte populations suggesting a broader role of FGF23 in the regulation of the immunological and inflammatory response. For example, FGF23 might alter monocyte function by inhibiting 1-α-hydroxylase and thereby 1,25D synthesis (244, 245). Furthermore, FGF23 can stimulate TNFα expression in macrophages (215, 216) (Figure 1). FGF23 effects on monocytes and macrophages seem to be mediated by Ras/MAPK signaling and occur in the absence of klotho (215, 244). FGFR1 is expressed at highest levels in these cells (216, 244) and, therefore, FGFR1 might mediate FGF23 effects. However, animal studies with monocyte/macrophage-specific deletion of FGFR1 will be necessary in order to determine if FGF23 can directly target these leukocyte populations. Since 1,25D can inhibit FGF23-induced TNFα in macrophages (215), it is interesting to speculate that FGF23’s inhibitory actions on the innate immunity can be counter-regulated by 1,25D (246), which would explain the stimulating effects of 1,25D on antibacterial macrophage response (247).

## Indications that FGF23 Might Affect a Variety of Other Tissues and Cell Types

While the effects of klotho and sKL on the central nervous system have been extensively studied, as reviewed elsewhere (77, 248), it is less clear whether FGF23 can directly target neurons. A study in hippocampal neuron cultures isolated from mice shows that FGF23 treatment reduces the complex cell morphology and enhances synaptic density (106). This effect occurs in the absence of klotho that is not expressed in hippocampal neurons, requires FGFR activity and involves PLCγ signaling. Co-treatment with sKL inhibits the FGF23 effect and causes an activation of Akt signaling (106). As Akt can be activated *via* FRS2α, this finding supports the hypothesis described earlier, that sKL might modify FGF23-induced downstream signaling (Figure 3). By directly targeting hippocampal neurons, FGF23 might contribute to learning and memory deficits which are observed in many patients with CKD, especially in children (249–251). Indeed, it has been reported that elevations in serum FGF23 levels are associated with cognitive impairment in CKD patients (252). Furthermore, transgenic mice overexpressing cleavage-resistant FGF23 resulting in elevated serum FGF23 levels show reduced long-term potentiation in the hippocampus and impaired spatial learning and memory (253). However, since administration of a high phosphate diet ameliorates this phenotype, FGF23-induced hypophosphatemia rather than direct FGF23 actions on the brain might be involved. Clearly, more detailed *in vitro* and *in vivo* experiments are necessary to test the existence of direct pathologic actions of FGF23 on neurons as well as other cell types in the central nervous systems.

Since FGF23 can directly induce injury of the heart muscle, it is tempting to speculate that FGF23 might also contribute to skeletal muscle dysfunction and atrophy that is found in many patients with CKD (254). Interestingly, animal models for ARHR and XLH with primary FGF23 elevations show deficiencies in skeletal muscle fiber contraction and develop muscle weakness, which is ameliorated after injections of an FGF23-blocking antibody (196, 255). FGF23 might also have beneficial effects on skeletal muscle, as elevations of circulating FGF23 by intraperitoneal injections of recombinant FGF23 in wild-type mice extends exercise performance (256). Similar to cardiac myocytes, skeletal muscle cells express FGFR4, but lack klotho (205), and it has been shown that FGFR4 is a key regulator of myogenic differentiation and muscle regeneration after injury (257, 258). Furthermore, activating FGFR4 mutations contribute to rhabdomyosarcoma, a childhood cancer originating from skeletal muscle (259). However, a recent study shows that acute and prolonged FGF23 treatments have no effect on the function of isolated mouse skeletal muscle fibers or on an established cell culture model for myoblasts and myotubes (260).

Several types of lung injury are associated with CKD (261, 262), raising the question whether serum FGF23 can directly target and damage the lung. Since kidney, lung, and heart are in close interactions with each other (263), it is possible that FGF23 serves as one of the many pathophysiologic factors that can impact the balance between the three organs. The lung expresses all four FGFR isoforms (198), and like the liver, the lung has high levels of FGFR4 (264). However, as global FGFR4 knockout mice do not develop a lung phenotype (198), the role of FGFR4 in the regulation of lung development and function is unclear. Furthermore, analyses of klotho expression in the lung have provided conflicting results (65, 75, 205, 265), and, therefore, it is unclear whether direct FGF23 actions would be klotho-dependent and/or klotho-independent. So far only one mechanistic study has aimed to analyze potential direct effects of FGF23 on lung cells (265). FGF23 can target bronchial epithelial cells *via* FGFR1 and klotho to induce Ras/MAPK signaling and the expression of the inflammatory cytokine IL-8, which occurs in concert with TGFβ signaling that potentiates FGF23 effects by elevating FGFR1 expression. Addition of sKL attenuates FGF23 actions (265), indicating that membrane-bound klotho and sKL have opposite effects in this scenario and again pointing toward counterbalancing protective effects of sKL in regards to FGF23’s pathologic actions. Interestingly, the FGF23 effect only occurs in bronchial epithelial cells isolated from patients with cystic fibrosis (265), suggesting that the priming of cells by disease-specific stimuli might be necessary for FGF23 responsiveness. Since patients with cystic fibrosis have elevated serum FGF23 levels (265), it is possible that by targeting the lung and other tissues, such as the liver (163), FGF23 contributes to the systemic inflammation that is common in this disease (266). It has also been speculated that FGF23 might serve as a biomarker for chronic obstructive pulmonary disease (267), and smokers have elevated serum FGF23 levels (268, 269), indicating that FGF23 might be involved in a wider spectrum of chronic lung disorders. Of note, mice with global FGF23 deletion develop lung emphysema, which can be partially rescued by the deletion of NaPi-2a resulting in a normalization of phosphate metabolism (270). This finding indicates that in this animal model lung injury might be caused by hyperphosphatemia and FGF23, suggesting important physiologic actions of FGF23 on the lung. While to date only little is known about potential effects of FGF23 on the lung, several human, and experimental studies have reported reductions in levels of klotho and sKL in a variety of lung disease, indicating cell-protective effects of sKL, as reviewed elsewhere (271, 272). Furthermore, mice globally lacking klotho develop pulmonary emphysema (65, 273, 274).

Whether the endothelium expresses klotho, or not, is currently under debate (275), and, therefore, it is unclear if FGF23 could have klotho-independent actions on blood vessels. Studies in cultured endothelial cells have shown that FGF23 can promote oxidative stress (92) and induce the expression of cell adhesion proteins (231), suggesting that FGF23 might contribute to endothelial dysfunction. However, clinical and experimental studies have shown that FGF23 does not associate with and contribute to vascular calcification in CKD (276), where rather elevations in serum phosphate levels act as the major culprit (177). Whether FGF23 affects other aspects of vascular injury associated with CKD and might directly target different cell types in blood vessels, such as vascular smooth muscle cells, needs to be determined. Similarly, it is currently investigated, whether FGF23 can directly affect bone cells in a paracrine manner, and if so, whether this effect requires klotho or not. Interestingly, it has been shown that FGF23 can directly target chondrocytes which lack klotho, and thereby suppress proliferation and induce hypertrophy and differentiation (159, 277, 278). As briefly mentioned before, also macrophages and monocytes (215, 216, 244, 245) as well as cells in the spleen (221) can respond to FGF23. Furthermore, FGF23 has been shown to increase proliferation of prostate cancer cell lines (279).

It has been shown that “classic” FGF23 target organs which express klotho under normal conditions can also respond to FGF23 in a klotho-independent manner. For example, FGF23 effects in the parathyroid gland are mediated by FGFR1 and klotho, resulting in FRS2α/Ras/MAPK signaling and reduced PTH secretion (15, 154) (Figure 1). Interestingly, in mice lacking klotho specifically in the parathyroid gland, FGF23 retains its inhibitory actions on PTH secretion, but activates calcineurin/NFAT instead of Ras/MAPK signaling (16). This animal model supports the hypothesis discussed above, that klotho determines the branch of FGF23-mediated downstream signaling (Figure 3). However, since both, klotho-dependent and klotho-independent signaling, have the same physiologic effect, i.e., to reduce PTH secretion, the biological relevance of klotho-independent signaling is unclear, but it might act as back-up mechanism to ensure FGF23 responsiveness of the parathyroid gland in situations of klotho deficiency. FGF23 seems to also affect the kidney in a klotho-independent manner, at least in the scenario of kidney injury. It has been shown in an animal model of acute tubulointerstitial injury that FGF23 contributes to fibrosis (190, 280) (Figure 1). However, FGF23 can only directly target fibroblasts that are derived from an injured kidney (190), indicating that pathologic actions of FGF23 depend on the context and the presence of other pathologic stimuli. FGF23 effects on injury-primed fibroblasts are mediated by FGFR4 and calcineurin/NFAT signaling and result in an upregulation of TGFβ production, which then carries the pro-fibrotic signal forward to induce extensive fibrosis and tissue injury (191). Since neither healthy nor injured renal fibroblast express klotho (190), this pathologic effect of FGF23 appears to be klotho-independent. Interestingly, the introduction of klotho expression or treatment with sKL causes a switch from calcineurin/NFAT to Ras/MAPK signaling in isolated injured renal fibroblasts and attenuates FGF23’s pro-fibrotic actions (191). Overall, this study supports the hypothesis that sKL can counterbalance FGF23’s pathologic effects by inducing a switch in FGF23-induced downstream signaling toward the FRS2α/Ras/MAPK cascade (Figure 3). Whether FGF23 can induce renal injury by targeting other cell types than fibroblasts as well as the spectrum of renal diseases that might involve FGF23 as a causative kidney-damaging factor remains to be established. Furthermore, it needs to be studied whether in “classic” FGF23 target organs, klotho-independent signaling only occurs in the situation of klotho reduction or absence, or can also co-exist in the same tissue or even cell in parallel to klotho-mediated signaling events and might, therefore, have physiologically relevant functions.

## Conclusion and Outlook

FGF23 can directly affect several cell types and tissues. Depending on the target, FGF23 actions occur in the presence or absence of klotho, require different FGFR isoforms and are mediated by different signal transduction cascades. The variety of molecular pathways that can be activated by FGF23 in a cell type-specific context ensures that FGF23 has numerous effects that differ among tissues, and can include changes in the cellular uptake and secretion of other factors as well as modifications of cell growth and migration. Klotho-independent FGF23 actions might be more widespread than originally thought. However, based on the diverse nature of FGF23-induced signaling events and effects, traditional read-outs to detect FGF23 responsiveness are not adaptable to every potential target tissue, thereby complicating the identification of novel FGF23 actions. Furthermore, *in vitro* studies that are necessary to determine potential direct effects of FGF23 on a defined cell type, are hindered by the fact that FGF23 requires specific signaling receptors and mediators whose expression levels, localization, or activity might change when cells are isolated and kept in culture over time. Current findings indicate that klotho-independent actions of FGF23 might be exclusively pathological. However, more animal experiments analyzing FGF23 effects in a concentration- and time-dependent manner are needed to confirm this view. It is also possible that such studies will reveal that initial FGF23 effects are cell protective and meant to compensate for tissue injury. Most likely, FGF23’s pathologic actions occur in combination with other injury and stress stimuli, whose presence might alter the “molecular make-up” of cells and thereby increase their FGF23 responsiveness. However, it will be challenging to mimic such a multifactorial scenario in cultured cells in order to test this hypothesis.

Besides osteocytes in the bone, several other tissues and cell types have been reported to produce FGF23. The original studies reporting the cloning of FGF23 include FGF23 expression analyses of selected human and mouse tissues by reverse transcription PCR, showing signals for heart, liver, brain, small intestine, lymph node, and thymus (10, 18). This outcome overlaps with more comprehensive qPCR-based tissue screens in mice, reporting that besides these tissues also spleen, lung, skeletal muscle, and stomach contain FGF23 mRNA (205, 281). More detailed analyses of tissues and cultured cells by qPCR, immunoblotting, and immunohistochemistry, have confirmed and extended the findings of these screening studies, showing FGF23 expression in the heart (184, 185, 192, 193, 282), liver (283, 284), kidney (190, 285–287), spleen (216, 288, 289), brain (18), skeletal muscle (256), bone marrow (216, 290–293), and macrophages (215, 294). Also certain types of tumors have been shown to produce FGF23 (10, 279). Combined with the various FGF23 effects, as described above, these findings indicate that FGF23 might not only act as a hormone, but also functions as a paracrine factor. Furthermore, FGF23 might play important roles during embryonic development. In zebrafish embryos, FGF23 expression is confined to the cells of the corpuscles of Stannius which regulate mineral ion homeostasis in advanced bony fish, also called teleosts (295). During embryonic mouse development, FGF23 is predominantly expressed in somites, heart, and liver (296). Although global FGF23 knockout mice are born at normal Mendelian ratios and do not develop an obvious phenotype before 10 days after birth (296), indicating that FGF23 is not essential for embryonic development, it is still possible that FGF23 contributes to proper organogenesis.

FGF23 expression often seems to occur in the context of pathologic stimuli or tissue damage, suggesting that either FGF23 contributes to the injury process or is induced to protect from injury. Tissue-specific knockout studies will be required to analyze the role of locally produced FGF23 in comparison to bone-derived, circulating FGF23. Furthermore, it needs to be determined whether the mechanisms that regulate FGF23 synthesis as well as posttranslational modifications and processing in the bone are also active in other FGF23 producing organs, or whether FGF23’s precise form and modifications and, therefore, bioactivity varies and depends on the source. Clearly, a key open question that needs to be answered is whether the N- and C-terminal FGF23 cleavage fragments have a biological function, and if so, whether this is different from the role of intact FGF23.

## Author Contributions

BR designed and generated the three figures. CF wrote the majority of the text, which then was edited by BR.

## Conflict of Interest Statement

Both authors declare that the research was conducted in the absence of any commercial or financial relationships that could be construed as a potential conflict of interest.
